# Combined Individual Experience and Accelerometry Measurement of Upper Limb Use in Daily Activities in Real Time After Stroke

**DOI:** 10.3390/s25237330

**Published:** 2025-12-02

**Authors:** Isuru Senadheera, Prasad Hettiarachchi, Brendon Haslam, Rashmika Nawaratne, Michael Pollack, Susan Hillier, Michael Nilsson, Damminda Alahakoon, Leeanne M Carey

**Affiliations:** 1 Centre for Data Analytics and Cognition, La Trobe Business School, La Trobe University, Melbourne, VIC 3086, Australia; i.senadheera@latrobe.edu.au (I.S.); p.hettiarachchige@latrobe.edu.au (P.H.); b.nawaratne@latrobe.edu.au (R.N.); d.alahakoon@latrobe.edu.au (D.A.); 2 Occupational Therapy, School of Allied Health, Human Services and Sport, La Trobe University, Melbourne, VIC 3086, Australia; b.haslam@latrobe.edu.au; 3 Neurorehabilitation and Recovery, The Florey, Melbourne, VIC 3086, Australia; 4 Centre for Rehab Innovations, The University of Newcastle, Callaghan, NSW 2308, Australia; m.pollack@newcastle.edu.au (M.P.); michael.nilsson@newcastle.edu.au (M.N.); 5 Hunter New England Health, New Lambton, NSW 2305, Australia; 6 Allied Health and Human Performance, University of South Australia, Adelaide, SA 5001, Australia; susan.hillier@unisa.edu.au; 7 College of Health, Medicine and Wellbeing, University of Newcastle, Callaghan, NSW 2308, Australia

**Keywords:** activity participation, experience sampling method, accelerometry, upper limb function, stroke rehabilitation

## Abstract

Use of the upper limb to engage in everyday activities is a key indicator of functional recovery of stroke survivors. In addition to functional capacity, personal and environmental factors contribute to real-world upper limb use post-stroke. We aimed to combine data from the experience sampling method (ESM), a method used to capture real-time engagement in daily activities, with accelerometry, an objective measurement of arm use, to evaluate arm use behaviours of adult stroke survivors living in real-world environments. Thirty mild–moderately impaired stroke survivors and 30 age-standardized healthy individuals were monitored over 7 days, using accelerometers on both wrists and four ESM beeps per day to capture individual experiences in daily activities. Stroke survivors showed significantly lower use of the affected arm across all activity domains compared to the non-dominant arm of healthy participants and reported perceived lower skill and higher challenge levels. Physical context, motor capabilities and activity type were associated with affected arm use behaviour, with greater use observed during social settings and in physically demanding tasks. These findings demonstrate that combining ESM with accelerometry provides a novel, ecologically valid framework to capture and interpret the interplay between capacity, context, and behaviour in everyday life. This approach offers opportunities to design personalized, context-aware rehabilitation strategies that promote meaningful functional reintegration after stroke.

## 1. Introduction

Functional limitation of the upper extremity is one of the most common disabilities after stroke [1]. Upper limb (UL) functional use is an important goal of stroke rehabilitation [2], with the assumption that arm capacity improvement in the clinic can enhance everyday function and independent living in the real-world environment. Performing in daily activities, or “doing”, is a key indicator of post-stroke recovery, as it supports overall well-being by enhancing feelings of independence and capability [3]. To participate in the activities that a person needs and wants to do in a meaningful way, an individual requires the necessary performance capacity and skills [4].

Neurological capacity measured by clinical assessment in the clinic may not guarantee that this capacity can be used in daily life in the person’s own environment. For example, one study reported that 52% of stroke survivors did not use their affected hand in performing daily activities at home, even though their motor capabilities were demonstrated in the rehabilitation clinic [5]. Moreover, stroke survivors’ perceptions of their recovery and participation can diverge from clinicians’ assessments, consistent with an underappreciation of the importance of psychosocial factors impacting daily arm use behaviour. As the idiom “use it or lose it” implies, not using the affected arm daily after a stroke will likely lead to further functional decline. The learned non-use phenomenon exhibited by some stroke patients is an example of “lose it”—with no or seldom use of the affected arm during their daily activities, although they might preserve the physical capacity to do functional tasks [6]. This reflects a “translation gap” between clinics and the person’s home that is commonly observed [7].

Using only clinical assessments to confirm the generalization—automatic translation of motor capacity improvement observed in the neurorehabilitation clinic to meaningful participation in real-world activities—is difficult and limited. This translation gap between what individuals can physically do and what they actually do is critical to understanding recovery outcomes in post-stroke rehabilitation. Personal factors, including motivation, confidence, self-perceived skill, satisfaction, meaning and challenge levels during task performance, contribute to meaningful activity participation [4,8]. This may also contribute to motivation and the experience of “flow” [9]. The flow experience, often regarded as a state of optimal engagement and enjoyment during an activity, is defined as the psychological state where individuals fully immerse themselves in an activity, leading to increased satisfaction and positive emotions [10]. These psychosocial factors will not only enhance the translation of neurobiological capacity improvements, but also adherence to rehabilitation protocols at home.

The Person–Environment–Occupation–Performance (PEOP) model [11], a widely used occupational framework, further informs such factors and relationships that contribute to the occupational therapy lens on human performance (“doing”), participation, and well-being. The PEOP model depicts the transactional nature of a narrative, both in these personal and environmental factors and in everyday activities. Therefore, it is vital that rehabilitation efforts consider not only the physiological improvements but also the contextual and psychosocial factors that influence daily use of the affected arm in real-world situations.

To design interventions that target effective translation of functional capacity improvements into meaningful daily use, there is a need to understand arm use outside therapy early in rehabilitation. Accelerometry has been found to be a tool to measure UL use in people with stroke and distinguish between affected and unaffected limbs [12]. An accelerometer is a sensor device designed to directly and objectively measure real-world arm physical activity [13,14,15]. The advantages of the arm accelerometer include the ability to unobtrusively record continuously for days and to measure the participant’s activity in a real-world environment, allowing continuous measurement of arm movement without requiring intensive supervision. It is a valid and reliable instrument that has been used to monitor UL activity in stroke survivors at different stages and with varying levels of severity [12]. It provides objective measures of physical activity, allowing clinicians to monitor and quantify arm movements, which can be particularly beneficial for tailoring rehabilitation interventions that focus on restoring functional independence and quality of life post-stroke [16]. This quantitative data can efficiently complement traditional subjective and clinical assessments of activity levels and participation, leading to a more holistic understanding of a stroke survivor’s recovery journey.

Despite these advantages, notable limitations are associated with the use of accelerometry in monitoring real-world UL use. One critical limitation is its inability to capture the activity context, including psychosocial factors [7,17]. Without contextual information about the UL functions being performed, assessments derived from accelerometry can fall short of providing a complete picture of the actual arm use behaviour.

The experience sampling method (ESM) [18], also known as ecological momentary method or ecological momentary assessment [19], is a valid and reliable tool used for capturing real-time experiences, to understand dynamic aspects of daily life and engagement in meaningful activities [20]. Such a real-time approach significantly reduces recall biases and increases ecological validity compared to traditional retrospective assessments [21]. In contrast, laboratory-based tests conducted in a controlled setting typically only provide a snapshot of functioning at a specific time, and thus lack ecological validity [22]. ESM allows therapists to gather data on participants’ thoughts, perceptions, feelings, cognitive functioning, and behaviours in their natural environments, enhancing the ecological validity of stroke rehabilitation strategies [23,24]. For example, Forster et al. employed ESM to conduct a microanalysis of mood and self-reported functionality in stroke patients, revealing a critical interplay between mood states and functional rehabilitation [25].

The insights gained through ESM tie closely to principles derived from flow theory [26], which reflects the individual’s choice of using his/her affected arm in activities that are challenging yet achievable. Specifically, in occupational therapy, adaptation or function is primarily concerned with the individual and their intense engagement in occupation, commonly referred to as the “just right challenge” [23]. Overlaying information of psychosocial contextual factors influencing real-world UL behaviours onto accelerometer measurements can highlight barriers and facilitators to arm use in activities.

The fusion of ESM (perceived performance during real-world activities) and accelerometer (actual arm movement) data presents an emerging approach that enhances the understanding of real-world arm use. The combination of objective measurements through accelerometry and subjective individual experiences through ESM has been found to be a feasible and valid method [7,22]. This approach allows a more contextualized view of daily UL behaviour, thus offering valuable insights into the factors that promote or hinder the automatic translation of neuro-biological rehabilitation outcomes into everyday activity participation. For example, in an accelerometry–ecology combined analysis, Chen et al. [17] highlighted that psychosocial aspects, such as self-efficacy and social context (alone or not), play a significant role in daily arm use, suggesting tailored rehabilitation strategies should engage stroke survivors holistically and address their biopsychosocial rehabilitation needs. However, they targeted only the affected arm use. A further recent study examined the feasibility, validity and acceptability of using integrated ESM and accelerometry measurement for monitoring daily activity participation and health-related symptoms among community-dwelling stroke survivors [22]. To the best of our knowledge, a combined analysis that integrates the self-perceived skill and challenge of performing daily activities (linked to flow concepts) in real time and objective measurement of bilateral arm movement and use has not been undertaken.

To build upon current research that addresses everyday activity participation (i.e., what participants do and how well they do it), we focused on chronic stroke survivors who have returned home, a time when long-term rehabilitation outcomes and quality of life are critical. The primary objective of this study was to fuse accelerometry measurements with self-reported experiences in performing activities in real time to characterize UL use in activities that community-dwelling chronic survivors of stroke choose to do. Through contextualized real-world arm use measurements, we aim to investigate the relationship between UL use behaviours, activities engaged in, and perceived challenges and skills in activity participation.

Secondly, we sought to characterize how healthy adult individuals perceive engagement in different activities and how they proportionately use their UL in those activities. This is critical to help interpret and benchmark post-stroke activity performance.

Lastly, we aimed to explore the momentary effects of social–environmental context and psychological factors (perceived skill-challenge, motivation and self-efficacy) that influence arm use and activity participation. Together, these insights provide a strong foundation for personalized rehabilitation.

## 2. Methodology

### 2.1. Study Design

Our study was an observational cohort study with a total sample size of 60 participants across two groups: 30 healthy adults/older adults and 30 adults/older adult stroke survivors [27]. A healthy control group was sampled using stratified criteria based on decade age and sex, ensuring an even distribution across decades. Stroke survivors were 3 to 18 months post-stroke and living in the community. The study was conducted with participants in their real-world environments.

Participants were required to wear wrist sensors and respond to questions from the custom-designed experience sampling mobile application known as “Staying Connected” [24] over each 7-day monitoring period. The experience sampling app was installed on their smartphones. Both healthy and post-stroke participants were asked to wear an accelerometer sensor on each wrist over the monitoring period, with exceptions allowed for swimming, showering and sleeping, while also responding to four random beeps from the experience sampling app. During the pre-monitoring visit, post-stroke participants completed screening and assessments, including the Action Research Arm Test (ARAT) [28], the National Institutes of Health Stroke Scale (NIHSS) [29], and the Stroke Impact Scale (SIS) [30].

### 2.2. Participants

We selected the healthy control group as follows: participants between 40 and 90 years of age with no self-reported diagnosis of any neurological conditions; no self-reported history or clinical evidence of cognitive impairment; capable and willing to use a mobile device for the duration of the study; and able to provide informed consent. Healthy participants were recruited in Melbourne, Victoria, between September 2023 and March 2025.

The selection of the stroke survivor group was as follows: stroke survivors who were (a) 18 years of age or older; (b) with clinical diagnosis of stroke confirmed by a medical professional; (c) stroke onset between 3 and 18 months prior to study enrolment; (d) medically stable, as determined by their physician, and not requiring hospitalization for stroke-related issues; and (e) able to provide informed consent. We excluded individuals with (a) current medical instability requiring hospitalization or precluding participation in the study; (b) severe unilateral spatial neglect impacting ability to use the mobile application; (c) prior history of other central nervous system dysfunction (e.g., traumatic brain injury and multiple sclerosis) that could confound the study findings; (d) severe peripheral neuropathy in the UL impacting ability to use the mobile application. Participants were recruited from three sites across Victoria, New South Wales, and South Australia. These individuals were recruited as part of the larger clinical trial known as TAILOR and CONNECT (ANZCTR registration number: ACTRN12623000666628).

### 2.3. Instruments

#### 2.3.1. Wearable Sensors

ActiGraph’s GT9X Link^TM^ and GT3X-BT^TM^ sensors developed by ActiGraph LLC, Pensacola, FL, USA [31] captured participants’ real-time UL movements during the 7-day monitoring period. Wrist acceleration along the 3 axes was sampled at a 60 Hz rate.

#### 2.3.2. Experience Sampling Mobile App

**Figure 1:**
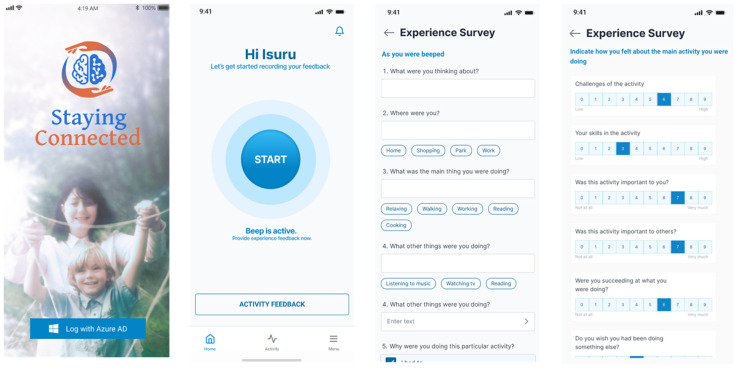
Screens from the Staying Connected experience sampling mobile application. The mobile app was made available to download from both Apple and Android app stores.

The “Staying Connected” experience sampling mobile app [24] was developed for this study to collect real-time experiences of participants’ participation in randomly sampled activities and their perceived challenges and skills in performing those activities. This app was able to send programmed beeps, displaying the experience sampling questionnaire, and saving participants’ responses along with timestamps. The experience sampling questionnaire was a modified version of the experience sampling questions based on Csikszentmihalyi et al. [18,32]. The questionnaire included a total of 35 brief questions: 2 questions regarding the main and secondary activities engaged in, 3 questions on physical/social context, 30 questions related to psychosocial–contextual information (including 2 for skill and challenge, 4 for self-efficacy, and 5 for activity and motivation). Permission to use the questions in the Staying Connected app for the period was obtained from the licence owners. Participants were beeped at 4 random times throughout the day, requesting them to record random activity experiences. The four beeps were scheduled during morning (09:30–11:00 h), midday (12:30–14:00 h), afternoon (15:30–17:00 h) and evening (19:00–20:30 h), and were set to expire after 90 min. Figure 1 shows some sample screens from the Staying Connected app.

### 2.4. Data Analysis

#### 2.4.1. Wearables

The raw accelerometer data were downloaded from sensors and pre-processed using ActiLife Software (version 6.13.5; ActiLife Corp., Pensacola, FL, USA). The software band-pass filtered data from each axis (x,y,z) between frequencies of 0.25 and 2.5 Hz to retain physiologically relevant movement frequencies while filtering out noise, and used a proprietary algorithm to calibrate acceleration due to gravity. The sensor data was then down-sampled to 1 Hz (non-overlapping 1 s epoch), and lastly converted acceleration into activity counts, representing summed activity counts per second [33,34]. Activity counts are defined as the sum of acceleration signals over a pre-determined epoch or period of time (1 activity count = 0.01664 g for an acceleration produced by a movement with 0.75 Hz frequency) [7,35]. Activity count vector magnitude (VM) was then calculated by compositing values from the 3-axis activity counts for every 1 s epoch (i.e., $\sqrt{x^{2}+\;y^{2}+\;z^{2}}$). Three-axis activity counts, and vector magnitude was smoothed using a 5-sample moving average to reduce the variability [36].

Non-wear times for the wearable sensors were determined using the Choi algorithm [37]. Wear times were then classified as sleep or wake time using the Sadeh algorithm [38]. Both non-wear and sleep times data were excluded from the dataset because they represent times when the sensor was not capturing true physiological movement or when arm movements during sleep are involuntary and not indicative of functional UL use in daily activities. A threshold of activity count ≥2 was used to define if the arm was moving or not at a given second [7,39]. When the activity count was <2, the arm was defined as not moving at a given second. This activity count threshold is commonly used in accelerometry studies to categorize epochs into arm movement vs. no movement [16].

The arm movement accelerometry signal was used to calculate the following indices: (1) amount of arm use, (2) ratio of arm use (asymmetry), and (3) arm use duration and ratio. The amount of arm use, inclusive of both spontaneous and voluntary movements, was calculated using the vector magnitude of the activity counts per second for the non-dominant upper limb for healthy controls and for the affected limb for post-stroke participants. The non-dominant limb of healthy individuals was selected as the control comparison to establish a baseline for normal function in a less-skilled limb, consistent with current literature. The symmetry of arm use was calculated as the asymmetry index = (activity of dominant or unaffected hand − activity of non-dominant or affected hand)/(activity of dominant or unaffected hand + activity of non-dominant or affected hand) × 100 [40]. Duration of arm use was computed by summing the seconds of arm movement, as defined through the ≥2 activity count threshold for each arm. The use ratio, which reflects the amount of time the non-dominant or affected arm is active relative to the amount of time the dominant or unaffected arm is active, is then calculated as the ratio between non-dominant or affected arm use duration and dominant or unaffected arm use duration [35].

The dataset was examined using descriptive statistical analysis to determine the frequency and patterning of arm use in everyday activity participation for both healthy and post-stroke groups.

#### 2.4.2. Experience Samples

Data collected from the Staying Connected experience sampling app were analyzed to investigate everyday activity experiences over the 7-day monitoring period. Perceived skill level and challenge level were captured using 10-point Likert scale values, which were centred and standardized into z-scores of each individual’s own mean to relate to the 8 domains of flow [18,23]. The flow experience begins when challenges and skills, as recorded in an experience sample at a given time, are above the person’s mean challenge and skill [26]. Skill and challenge z-scores were mapped onto Csikszentmihalyi’s eight-quadrant diagram that represents the flow model of mental state, with skill vs. challenge level as the axes [41]. The proportion of experience samples that fell into the defined flow quadrant (high skill and high challenge) was considered a “flow” state, representing a “just right challenge”.

The ‘main activity’ item (“What was the main thing you were doing?”) was categorized based on an activity list that included 100 activities from the Activity Card Sort (ACS) version 3 [42], with 4 additional activities based on participant-reported main activities (i.e., rest and relaxation, having a cuppa, talking on the telephone, and talking with family and neighbours), and 4 activities of daily living (ADL) [43]. Sleep was excluded from rest and relaxation since sensor data were pre-processed to exclude sleep. Rehabilitation-related activities reported by the post-stroke group, including therapy exercises and visits to clinics, were grouped into “therapy/exercises”. Activities which did not match the ACS-based list were grouped into “other activities”. The main activities were then grouped into 5 domains: activities of daily living (e.g., eating and dressing), instrumental activities of daily living (IADL) (e.g., shopping and doing dishes), leisure activities (e.g., watching TV and going to the park), fitness/health activities (e.g., exercise and organized sports) and social activities (e.g., eating out and vising family), based on ACS domains [42].

Nine questions assessed self-efficacy, activity and motivation aspects. These semantic differential and 7-point Likert scale values were grouped to summarize the following two dimensions [18,23]: (a) activity and motivation: detached–involved, wishing to do something else, activity importance to self and others, and feeling good; and (b) self-efficacy: control over the situation, succeeding at activities, living up to own and others’ expectations. Z-scores of each individual’s own mean were then calculated for each question and averaged to derive each dimension score [23].

The participants’ living or social situation, assessed through the question “Who were you with?” was categorized as alone or not. The places where the activities took place, assessed by “Where were you?”, were categorized as “at home” and “not at home”.

**Figure 2:**
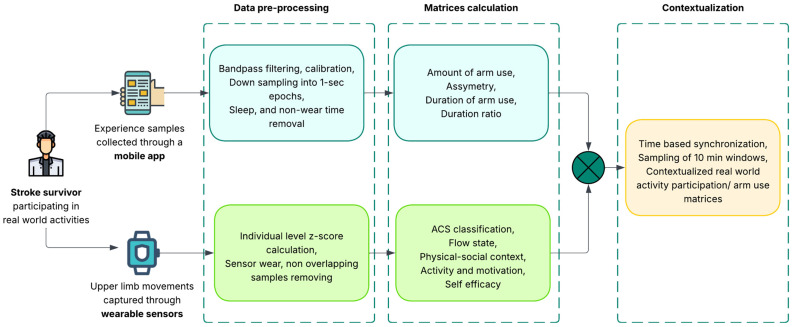
Data processing pipeline used to combine experience samples with wearable sensor data, contextualizing objective accelerometry measurements of real-world upper limb use in daily activities in real time.

Accelerometry data were then synchronized with experience samples using timestamps. A delineated 10 min time window of accelerometry data was synchronized, which was adjusted for 5 min and 3 min before the individual’s experience sample was recorded for post-stroke and healthy groups, respectively, to allow time to complete survey responses. The selection of a 10 min window was based on the literature reporting on physical activity levels of adults [7]. Based on our data exploration, we hypothesize that a post-stroke/healthy individual would take 5 or 3 min to complete the experience survey through the mobile app. Figure 2 shows the complete data processing pipeline.

### 2.5. Statistical Analysis

All descriptive statistics were computed using Python (Version 3.10). Correlation analysis was performed to investigate relationships among outcomes, followed by regression analysis to examine the predictive power. Due to the nature of the underlying distribution, the momentary effects of social context (e.g., alone vs. social; and home vs. not at home), psychological factors (perceived challenge vs. skill; self-efficacy; and activity and motivation), and motor capacity that influence affected arm use, were analyzed with generalized linear models using IBM SPSS Statistics version 30.

## 3. Results

### 3.1. Participant Characteristics

Thirty participants per group were included in the study (*n* = 60 total). Participants’ characteristics, including demographics for both groups and stroke-specific factors for the post-stroke group, are shown in Table 1. On average, post-stroke participants were 61.5 years of age, 13 months post-stroke (range 3 to 20 months), and distributed across sex (67% male), non-dominant-hand affected (53%), had mild-to-moderate stroke severity (NIHSS), moderately recovered (SIS perceived recovery), and poor to good functional arm capacity (ARAT) at the time of entry to the study. In contrast, healthy participants were an average of 59.9 years of age and 63% were females. Data from the age-standardized healthy control group were used as a benchmark for comparing arm use in the stroke survivor group.

### 3.2. Data Availability

A valid day of accelerometer wearing was defined as having at least 10 h of wearing during waking hours. All participants achieved 7 valid days of wear with average accelerometer wearing time of 13.0 and 13.2 h/day across 7 days for post-stroke and healthy participants, respectively. Data were considered valid when accelerometers were worn at the same time that experience samples were recorded. In total, 443 and 403 experience samples with valid accelerometry measurements for 30 individuals from post-stroke and healthy groups, respectively, were included in the analysis. From the total experience samples, 424 and 387 samples matched to ACS-based list of activities. The average experience sampling questionnaire completion rate per participant ranged from 57% to 67% for post-stroke and healthy groups, respectively. The average time to respond from beep time ranged from 12.7 to 10.6 min for post-stroke and healthy groups.

### 3.3. Activities Engaged in Real-World Context

**Figure 3:**
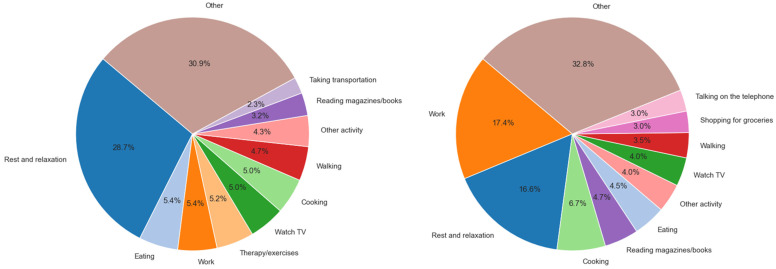
Participant-reported most frequent activities for post-stroke (**left**) and healthy (**right**) groups, as categorized with ACS. All non-frequent ACS activities were grouped into “other”. Activities which did not match the ACS-based list were grouped into “other activity”.

Figure 3 presents the most frequent activities that post-stroke and healthy groups chose to perform, as informed by our modified version of the ACS classification. Stroke survivors spent most of the time resting and relaxing (28.7%). In contrast, the healthy group participated most frequently in work (17.4%). Notable was the low percentage of work activities (5.4%) in the post-stroke group. Both groups showed similar levels of participation in ADLs such as eating (5.4% post-stroke and 4.5% healthy), IADLs such as cooking (5.0% post-stroke and 6.7% healthy), leisure activities such as watching TV (5.0% post-stroke, 4.0% healthy) and fitness/health activities such as walking (4.7% post-stroke and 3.5% healthy). Notably, the post-stroke group engaged more in sedentary activities, including sleep, and in therapy/rehab exercises (5.2%). There were fewer social activities observed in the top ACS observed for the post-stroke group. In comparison, the healthy group reported active engagements in physically demanding IADLs such as shopping for groceries, light cleaning, driving, and doing dishes, as well as frequent social interactions such as talking on the telephone, talking with family and neighbours.

### 3.4. Arm Use Behaviours Between Activities and Perceived Challenges and Skills in Activity Participation

**Figure 4:**
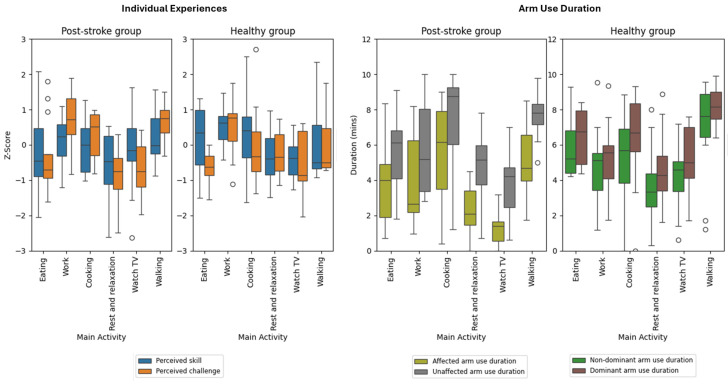
Participant-reported 6 ACS activities most frequently and commonly engaged in across groups and group-level perceived performance (skill and challenge) and arm use duration for each group (affected vs. unaffected for post-stroke, and non-dominant vs. dominant for healthy).

**Figure 5:**
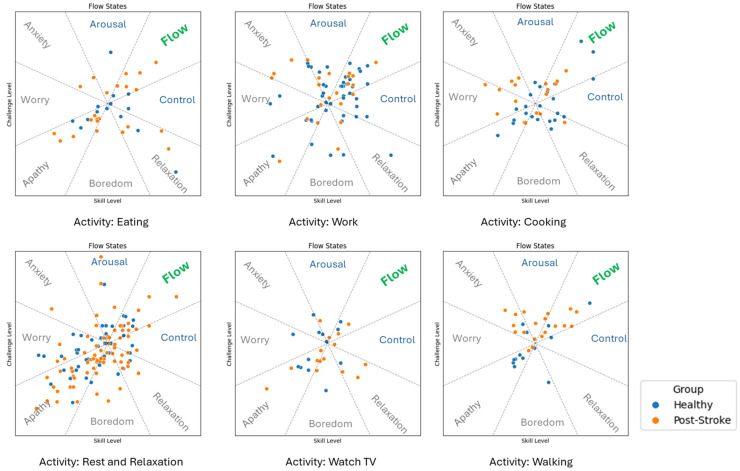
Self-reported perceived skills vs. challenge levels mapped into concepts of flow for the most frequent top 6 activities according to ACS-based classification. Orange and blue colours represent experience samples from post-stroke and healthy groups, respectively.

Figure 4 and Supplementary Table S1 show the objectively measured UL use and ESM perceptions in performing the most frequent six daily activities (selected taking into account both groups) and categorized according to the ACS. Figure 5 shows flow state diagrams for each activity reflecting the “just right challenge”. Post-stroke participants showed significantly lower skill and affected arm use duration during the task of eating (3.58 ± 2.21 min) compared to non-dominant arm use in healthy individuals (5.87 ± 1.84 min). While both groups experienced work with comparable flow levels (~33%), healthy individuals demonstrated higher skill ratings and slightly longer arm use.

Cooking was associated with one of the highest amounts of affected arm use (5.38 ± 3.03 min) in the post-stroke group, with a mean difference (increase) of 0.12 min compared to non-dominant arm use in the healthy group, though post-stroke participants reported higher challenge and lower skill. Notably, the post-stroke group was most actively engaged during cooking, reporting a median magnitude of 11.98 (IQR 22.29) of affected arm engagement compared to 14.08 (IQR 30.90) magnitude of non-dominant arm in the healthy group based on accelerometry measurement.

During rest and relaxation and watching TV, both groups reported low skill and challenge, but post-stroke participants showed higher flow during TV viewing (40.9%), despite minimal arm use, indicating passive UL engagement. In the health/fitness activity domain, walking showed significant active engagement; post-stroke participants reported higher challenge and flow (38.0%), with modest affected arm movement intensity (1.25 median VM), while healthy individuals had significantly greater movement intensity (49.87 median VM) in the non-dominant arm.

Across most frequent activities, post-stroke individuals generally showed lower skill, higher challenge, reduced arm use, and, in some contexts (e.g., watching TV, cooking), significantly higher flow (specifically in IADL and leisure activities) compared to healthy individuals. The most flow-inducing activities for the post-stroke group were watching TV (40.9%), walking (38%), cooking (36.3%) and working (33.3%). In contrast, the healthy group reported frequent flow when working (31.4%) and walking (28.6%).

### 3.5. The Momentary Effects of Social Context and Psychological Factors That Influence Affected Arm Use Relative to Perceived Participation, and in Relation to Individual’s Motor Capacity

#### 3.5.1. Group Level Analysis Between Post-Stroke and Healthy Groups

Objective measurements of arm use and perceived activity performance for each ACS category and person-environment activity context factors are shown in Table 2. The post-stroke group most frequently engaged in leisure activities (44.9%), 12.9% more than observed in the healthy group, and 15.5% less in IADLs (25.9%) compared to healthy individuals. Notably, slightly increased participation in fitness/health activities (11.3%) and ADLs (9.7%) was seen in post-stroke individuals compared to the healthy group.

Post-stroke individuals showed low affected arm use across all activity categories, with a median VM of 0.00 for ADLs and leisure activities, suggesting minimal active participation from the affected side in these tasks. Higher affected arm use was seen in social (3.04 median VM) and instrumental (1.40 median VM) activities, though still notably lower than for the unaffected arm. The asymmetry of arm use was highest during fitness activities (median = 29.81) and lowest in social activities (0.15), reflecting slightly more symmetrical use when engaging socially post-stroke.

In comparison, healthy participants displayed relatively symmetrical arm use across all activity types, with asymmetry medians close to zero. Arm use intensity was greatest in fitness/health, which is physically demanding (non-dominant arm median 26.84 VM), and instrumental activities (non-dominant arm median 16.18 VM), suggesting active engagement of both upper limbs in these activities.

Duration-based patterns of arm use aligned with intensity measures. Post-stroke participants used their affected arm for the shortest durations during leisure activities (2.68 ± 1.37 min) and longest during social activities (4.48 ± 1.97 min) and fitness/health activities (4.07 ± 1.88 min). The use ratio (affected/unaffected) was highest in social activities (0.75 ± 0.39), consistent with relatively more balanced intensity of arm movements shown during social engagement. In contrast, healthy adults showed balanced arm use durations, with the highest duration ratios in fitness/health (0.87 ± 0.29) and social (0.86 ± 0.17).

Post-stroke individuals perceived fitness/health activities as the most skillful and challenging, reporting the highest median skill (0.28) and challenge (0.89) levels. Motivation and self-efficacy were also highest in fitness activities. Instrumental activities showed moderate skill (−0.16) and high challenge (0.36) scores, while leisure activities were typically perceived as low in both skill (−0.29) and challenge (−0.56), with only 16.1% of these activities inducing flow state.

Among healthy participants, fitness/health activities and IADLs were similarly rated as higher in skill, challenge, and flow potential, with fitness/health producing the highest flow occurrence (27.6%) followed by IADLs (24.5%). Notably, most common flow-inducing activities in the post-stroke group were fitness/health (36%) and IADLs (26%), which aligned with the healthy group. Leisure activities of healthy individuals elicited the lowest challenge scores and had moderate flow reports (17.8%).

Activity context patterns highlighted that the post-stroke group spent 66% median time at home during fitness/health activities, which is a significant deviation compared to the healthy group. Moreover, the post-stroke group spent more time alone (median 23% more) at home (median 14% more) during leisure activities and more time “not at home” (median 21% more) during social activities than the healthy individuals.

#### 3.5.2. Group Analysis by Post-Stroke Functional Motor Capacity

When stratified by affected arm functional capacity (low, moderate, or good) based on ARAT scores (0–9, 10–56, or 57, respectively) [45], distinct differences in post-stroke activity engagement and arm use patterns emerged (Table 3).

Stroke survivors with low functional motor capacity showed passive engagement (characterized by minimal affected arm use) across most activity types, with median vector magnitude values at or near zero for ADLs, instrumental, and leisure activities. The highest activity engagement for this subgroup occurred in social activities (median 2.27 VM), followed by fitness/health (median 0.55 VM). Correspondingly, the affected arm was used least for leisure (mean of 2.16 min) and most for fitness/health (mean of 4.12 min). Notably, activities perceived as most skillful and highest challenge included fitness (skill: 0.54, challenge: 0.92) and instrumental (skill: 0.23, challenge: 0.36) activities. Leisure activities were consistently rated lower in both skill and challenge.

Those in the moderate capacity group showed greater affected arm engagement intensity in IADLs (median 1.83 VM) and social (median 2.47 VM) activities, with longer mean use durations across all categories compared to the low-capacity group; particularly in social activities (4.56 min) and ADLs (3.75 min), which is nearly 1 min mean increase than the low-capacity group. Comparatively, fitness/health and IADLs were perceived as higher in difficulty (0.87 and 0.56, respectively), while skill ratings were generally low to modest across categories, with fitness (0.28) perceived as the one requiring the most skill.

The good functional capacity group used their affected arm most intensively in IADLs (median 9.67 VM) and social (median 6.65 VM) activities, with the longest mean durations in social activities (6.06 min). This group perceived social activities as both highly skillful (1.54) and highly challenging (1.07) relative to the other groups. Interestingly, IADLs and social activities emerged as key domains for actively “doing” in those with greater functional capacity.

#### 3.5.3. Social Context During Activity Participation

**Figure 6:**
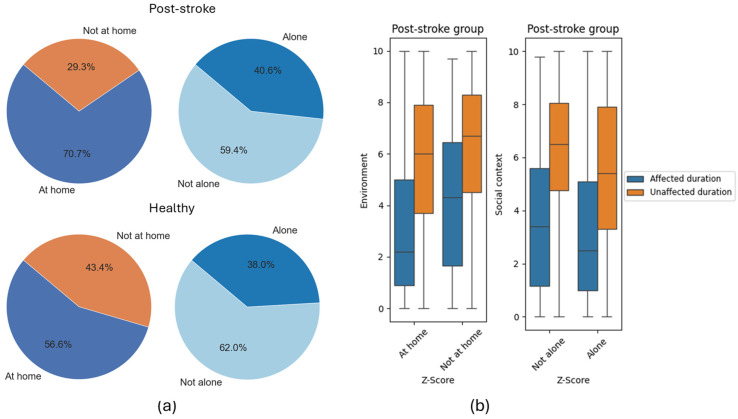
(**a**) Participant-reported environment (**left**) and social context (**right**) for post-stroke (**top**) and healthy (**bottom**) groups; (**b**) duration of arm use for affected and unaffected arms in different environments (home or not at home) and social contexts (alone or not alone) for post-stroke group level.

Figure 6 shows the social context of activities. Post-stroke participants spent most of the time at home (70.7%) compared to healthy individuals (56.6%). Median affected arm use duration increased by 2.1 min when not at home post-stroke. Furthermore, the time post-stroke participants spent alone (40.5%) is slightly higher than that of healthy individuals (38%) and showed an increased median use duration of the affected arm in social contexts (3.4 min) than when alone (2.5 min).

#### 3.5.4. Influence of Momentary Effects of Social Context and Psychological Factors on Affected Arm Use

Table 4 reports generalized linear regression analyses used to examine momentary effects of social context (e.g., alone vs. not alone, and home vs. not at home) and psychological factors (perceived challenge vs. skill, self-efficacy, and motivation) that influence arm use relative to perceived participation, in addition to an individual’s motor capacity, especially for affected arm use.

The ARAT score significantly predicted post-stroke affected arm use (Table 4 Model 1). The linear model (Model 1) was statistically significant (Omnibus χ^2^ = 4.138 and *p* = 0.042) and showed a reasonable fit (residual deviance/df = 1.791), accounting for approximately 5% of the variance. For every 1 point increase in ARAT score, the median affected arm use would increase around 17 min over a day (~11 h monitoring period per day).

Models 2 and 3, generalized linear mixed models, accounted for approximately 25% and 31% of the explained variances of affected arm use duration, respectively. During leisure activities, post-stroke participants used their affected arm, on average, 1.5 min less compared with other activity types (95% CI: −3.0 to −0.1, *p* < 0.05, Model 2) (Figure S1). If participants are not at home, they would use their affected arms approximately 70 min more than when they are at home (β = −1.075, 95% CI: −1.929 to −0.220, *p* < 0.05, Model 3, ~over 11 h monitoring period per day). The effect of motor capability on the affected arm further differed by physical context. For example, the more capable individuals used the affected arm more when not at home than when at home (ARAT × at home β = −0.47, 95% CI: −0.89 to −0.006, *p* < 0.05).

## 4. Discussion

In this study we provide novel insights into how chronic stroke survivors engage their affected upper limb in everyday life by integrating objective accelerometry with real-time subjective experience sampling. Unlike traditional laboratory or capacity-based assessments, our approach captures the interplay between what individuals can do, choose to do, and actually do across diverse real-world contexts. By combining behavioural data with perceptions of skill, challenge, and flow, we mapped arm-use behaviours as they unfold naturally within participants’ lived environments. Benchmarking these patterns against age-standardized healthy adults allowed us to describe distinctive post-stroke participation profiles—revealing not only reduced affected arm use, but also the influence of contextual, psychosocial, and task-related factors on engagement. Collectively, these findings highlight the potential of combined accelerometry–experience sampling to move beyond static measurement toward personalized, context-aware rehabilitation frameworks that more accurately reflect and target real function and participation in everyday activities.

The fusion of accelerometry and experience sampling data allowed the linking of objective arm use measurements to real-world activities performed and to subjective individual experiences. To the best of our knowledge, apart from two feasibility studies [7,22], only one study has reported results using such a method [17]. By contextualizing arm use within lived experiences, our approach moves beyond wearable sensor-only metrics and provides a more ecologically valid understanding of everyday activity participation. The use of the Activity Card Sort-based list further provided a structured framework for categorizing everyday activities. Reported ACS activities engaged in by survivors of stroke were consistent with the literature [46].

Our results highlight three key findings: (1) stroke survivors are less actively engaged and chose to do home-based activities more frequently, with lower engagement in work and social domains compared to healthy adults; (2) post-stroke affected arm use behaviours are heterogeneous across activities and influenced by motor capabilities; and (3) social context—particularly at home vs. not at home—is associated with affected arm use above and beyond motor capacity alone.

In addition, our results emphasize the influence of momentary effects on the “translation gap” between what stroke survivors can do and what they actually do in their daily lives. Even participants with good functional capacity (ARAT = 57) did not fully engage their affected arm in activities compared to healthy individuals, with notable underuse in sedentary contexts, such as in leisure activities and during certain ADLs. In contrast, certain activities—especially IADLs (e.g., cooking), as well as social and fitness/health (e.g., walking)—elicited higher affected arm engagement, suggesting that goal-directed, and moderately demanding valued activities may better translate functional capacity into real-world use.

### 4.1. Activity Domains and Potential Rehabilitation Targets

The post-stroke group spent nearly half of their sampled time in leisure activities, particularly rest and relaxation (28.7%). While leisure is an important contributor to quality of life, the predominance of low demand physical leisure may be a signal for low UL engagement in everyday life. Our findings parallel accelerometry-only studies showing low affected arm activity in home environments [33,47], but add novel context: these low-use periods are not simply the absence of movement—they are embedded in activity types and contexts that inherently offer fewer physical demands.

Notably, although cooking was perceived as more challenging and individuals reported they were less skilled in the post-stroke group, it elicited some of the highest affected arm use durations and magnitudes. Targeting such high-engagement activities in rehabilitation interventions at home could support more naturalistic, self-sustaining UL practice.

Social activities also stood out. While they accounted for a smaller proportion of sampled time post-stroke, they were associated with active engagement, more symmetrical arm use (median asymmetry 0.15, mean use ratio 0.75) and higher perceived skill (median z-score 1.54) among those with greater functional capacity. This reiterates that social interactions can stimulate more physical activity and increase engagement [48]. Though social context (alone or not) was reported as a significant factor for increased arm use in a similar combined accelerometer–experience sampling study [17], our study added further context into the activities participants engaged in. Rehabilitation strategies that incorporate social engagement—whether through family interaction or social activities—may therefore serve dual purposes: improved participation and greater arm use.

### 4.2. Intrinsic and Extrinsic Moderators

Beyond motor capacity, our regression models showed that motor capability, activity and physical context were significantly associated with the amount of affected arm use. Being not at home was associated with greater arm use, suggesting that social interactions, environmental demands and affordances (e.g., navigating social spaces) promote UL engagement. Similarly, social presence coincided with higher affected arm use, particularly in the good-capacity subgroup (mean duration 6.06 min). This may reflect increased self-presentation, cooperative task demands, or the natural physicality of social interaction.

The PEOP model [10] helps explain these findings and develop tailored therapy. While functional capacity (a person factor) is foundational, occupational performance and activity participation also depend on environmental opportunities (e.g., not being in the home environment vs. at home; alone vs. not alone/social), activity demands (occupation characteristics such as challenge and skill needed), and perceptions (psychology—“person” factor). As greater affected arm use was seen from higher functional capacity groups, complex IADL and social activities with greater social and environmental demands promoted activity performance. Low-capacity participants, by contrast, were more often in passive, home-based contexts, limiting both physical opportunities and motivational triggers for affected arm engagement. The PEOP model suggests that these ecologically valid interactions among such intrinsic, extrinsic factors and person–environment fit should be considered when tailoring therapeutic interventions to better promote translation of neurobiological capacity into real-world UL engagement.

Activities with higher challenge and skill ratings, such as fitness/health activities, tended to produce more engagement, consistent with “just right challenge” in occupational therapy—conceptualizing flow theory into the study of human occupations [23,26]. Our results support that achieving the “just-right challenge” in everyday activities, where tasks are difficult enough to engage attention and skill but not so hard as to cause frustration, may be key for eliciting motivated and sustained affected arm use. This concept could be applied in designing home-based therapy tasks [23], tailoring both activity goals (such as cooking) and their contexts to optimize reintegration into daily life post-stroke. In turn, this will help ensure that therapy planning and outcomes are not solely based on clinical assessments but also on real-world functioning and patient experiences.

Interestingly, some frequent flow-inducing contexts (e.g., watching TV) showed minimal arm movement, highlighting that flow does not inherently guarantee active physical engagement. Though the flow experience commonly occurs when an activity stretches the person’s capacity and provides a challenge to his/her skills, activities which appear passive, such as watching TV, could also lead to flow in certain contexts [49]. This nuance reinforces the value of contextualized measurement: accelerometry alone would not capture that participants felt engaged during low-movement activities, while ESM alone would not reveal the relative motor inactivity during those same periods.

Categorizing the post-stroke group by functional arm capacity (ARAT) revealed distinct participation and affected arm use patterns. Those with low capacity showed minimal affected arm active engagement across most activities, even when reporting high perceived skill (e.g., IADLs). Being able to align contextualized arm use with the flow concepts will be important for the therapist to understand such instances as potential motivators or anxiety-producing activities for a client. In contrast, moderate-capacity individuals demonstrated more balanced engagement in IADLs and social activities, and high-capacity individuals engaged most intensively in complex, socially embedded activities.

These gradients underscore the need for capacity-tailored interventions and activities. For low-capacity individuals, targeted support to integrate the affected arm into simple, repetitive, meaningful tasks may help build confidence and reduce learned non-use. For moderate- and high-capacity individuals, rehabilitation could focus on increasing exposure to complex tasks, such as cooking, work, shopping, and taking transportation in varied environments to consolidate and generalize skills.

## 5. Study Limitations

This study has several limitations. Firstly, our study included adults with mild to moderate stroke, limiting the generalizability of findings to those with severe stroke and older adults with fewer skills to use technology applications. Secondly, our sample size, while sufficient for detecting group-level trends, may limit power for detecting smaller interaction effects, particularly in regression models. Thirdly, accelerometry thresholds for defining “movement” (≥2 activity counts) may not capture fine motor activity or differentiate between functional and non-functional movements [50], like using the limb as a stabilizer, for example. Lastly, ESM relies on participant compliance and self-report, which may introduce response bias despite random prompting. Although we minimized recall bias through real-time sampling, participants may still have altered behaviour in anticipation of prompts (Hawthorne effect).

*Future Recommendations and Clinical Implications*

The fusion of accelerometry measurement of arm use and ESM has the potential to provide new insights into real-time everyday activity engagement and arm use linked with individual experiences. Future research could apply this combined approach longitudinally to monitor how capacity, context, and activity engagement interact over time. This approach provides a framework for future research with technology applications in tailoring rehabilitative interventions. For example, this combined method could be integrated into clinical mobile applications with therapist dashboards.

Modifiable factors, such as social engagement (away from home), which were associated with increased affected arm use, could be further explored as a means to encourage greater UL use among community-dwelling stroke survivors. Moreover, the use of flow theory as a motivation framework to promote UL engagement can be further explored for therapeutic application. Future studies could use ESM to investigate the nine characteristics of flow experience [10,51] in tailoring post-stroke rehabilitation interventions.

## 6. Conclusions

The fusion of data from the two methods used in this study provides new insights into arm use and individual experiences during everyday activity engagement post-stroke and can be used to monitor activity participation levels of community-dwelling stroke survivors. By combining accelerometry with ESM, we obtained a nuanced, ecologically valid understanding of post-stroke arm use that takes into account motor capability, activity type, social context, and psychological states. Social and environmental factors such as not being in the home environment, motor capability and activity type were associated with affected arm use. Stroke survivors’ real-world activity participation patterns reveal that high-capacity individuals engage more actively in complex, socially embedded tasks, while low-capacity individuals remain in passive engagements. Contextualized understanding of real-world UL use behaviours guides therapists to effectively address the translation gap from clinical to real-world outcomes, and to develop tailored, context-driven, evidence-based rehabilitation strategies maximizing both functional independence and quality of life.

## Figures and Tables

**Table 1:** Participants’ demographics and clinical characteristics.

Characteristic		Post-Stroke Group (*n* = 30)		Healthy Control Group (*n* = 30)	
		*n*	%	*n*	%
Gender	Male	20	67	11	37
	Female	10	33	19	63
Handedness	Right	24	80	27	90
	Left	6	20	3	10
Affected side	Dominant	14	47	n/a	
	Non-dominant	16	53	n/a	
		Mean ± SD		Mean ± SD	
Age	(years)	61.5 ± 13.0		59.9 ± 10.8	
Time since stroke	(months)	13.4 ± 4.6		n/a	
Upper limb functional capability (affected arm)	ARAT score (out of 57)	26 ± 20.6		n/a	
	Low *n* (%)	10 (33)			
	Moderate *n* (%)	17 (57)			
	Good *n* (%)	3 (10)			
Stroke severity	NIHSS score	4 ± 3		n/a	
Perceived recovery	SIS recovery score (*n* = 26)	44.8 ± 18.2		n/a	

Note: ARAT scores were categorized as follows: 0–9 low, 10–56 moderate, 57 good [44].

**Table 2:** Objective measurements of arm use, how participants perceive they are carrying out the tasks, and physical–social context factors for self-reported activities participants chose to do. Activities and individual-level measurements (mean/median) grouped by ACS activity domains between post-stroke and healthy groups.

		Activity Category (Based on Activity Card Sort Version 3) *				
		Activities of Daily Living	Instrumental Activities	Leisure Activities	Social Activities	Fitness/Health Activities
Post-stroke	*n* (%)	43 (9.7%)	115 (25.9%)	199 (44.9%)	17 (3.8%)	50 (11.3%)
Healthy	*n* (%)	27 (6.7%)	167 (41.4%)	129 (32.0%)	35 (8.7%)	29 (7.2%)
Amount of arm use (vector magnitude)		Median (IQR)				
Post-stroke	Affected	0.00 (3.44)	1.40 (16.51)	0.00 (0.00)	3.04 (3.99)	0.88 (9.58)
	Unaffected	23.44 (35.48)	18.52 (36.69)	7.15 (32.79)	14.42 (28.11)	28.87 (35.68)
	Asymmetry	26.10 (70.24)	28.38 (50.00)	15.17 (62.30)	0.15 (49.72)	29.81 (74.00)
Healthy	Non-dominant	1.70 (27.79)	16.18 (28.20)	0.00 (0.40)	8.91 (27.85)	26.84 (53.10)
	Dominant	19.52 (36.68)	25.36 (46.51)	1.40 (4.64)	19.45 (25.30)	35.98 (62.61)
	Asymmetry	0.00 (0.46)	0.00 (2.97)	0.00 (0.00)	0.00 (7.05)	0.00 (12.60)
Duration of arm use (minutes)		Mean ± SD				
Post-stroke	Affected	3.36 ± 2.44	3.73 ± 2.39	2.68 ± 1.37	4.48 ± 1.97	4.07 ± 1.88
	Unaffected	5.84 ± 2.52	5.90 ± 2.52	5.36 ± 1.28	6.09 ± 1.74	6.61 ± 1.42
	Use ratio	0.55 ± 0.35	0.57 ± 0.28	0.48 ± 0.22	0.75 ± 0.39	0.60 ± 0.25
Healthy	Non-dominant	4.20 ± 2.91	5.62 ± 1.75	3.72 ± 1.36	5.47 ± 1.92	6.04 ± 2.51
	Dominant	4.79 ± 2.86	6.49 ± 1.45	4.57 ± 1.67	6.39 ± 1.75	6.97 ± 1.79
	Duration ratio	0.72 ± 0.44	0.84 ± 0.22	0.86 ± 0.25	0.86 ± 0.17	0.87 ± 0.29
Perceptions (z-score)		Median (IQR)				
Post-stroke	Skill	−0.16 (0.99)	−0.16 (0.89)	−0.29 (0.77)	−0.05 (0.82)	0.28 (0.84)
	Challenge	−0.21 (1.33)	0.36 (0.87)	−0.56 (0.49)	−0.11 (0.91)	0.89 (0.97)
	In Flow state ***n* (%)**	9 (20.9%)	30 (26.0%)	32 (16.1%)	2 (11.8%)	18 (36.0%)
	Activity and motivation	0.04 (0.46)	0.24 (0.27)	−0.17 (0.42)	−0.00 (0.38)	0.37 (0.41)
	Self-efficacy	0.12 (0.85)	−0.07 (0.47)	0.06 (0.53)	0.15 (0.68)	0.26 (0.45)
Healthy	Skill	0.13 (1.30)	0.25 (0.75)	−0.11 (0.74)	−0.07 (1.09)	0.34 (1.44)
	Challenge	−0.58 (0.40)	0.29 (0.52)	−0.38 (1.00)	−0.25 (0.62)	0.42 (2.05)
	In Flow state ***n* (%)**	2 (7.4%)	41 (24.5%)	23 (17.8%)	4 (11.4%)	8 (27.6%)
	Activity and motivation	−0.11 (0.64)	0.16 (0.24)	−0.25 (0.42)	0.26 (0.34)	0.26 (0.56)
	Self-efficacy	0.15 (0.54)	0.22 (0.50)	−0.03 (0.61)	0.16 (0.60)	0.31 (0.71)
Physical-social context (%)		Median (IQR)				
Post-stroke	At home	100 (0)	62.50 (41.43)	84.62 (25.00)	25 (93.75)	66.67 (50)
	Alone	0 (50)	50 (56.67)	38.46 (76.39)	0 (0)	14.58 (91.67)
Healthy	At home	100 (50)	60 (34.66)	70 (28.33)	46.43 (80.83)	0 (53.12)
	Alone	0 (50)	45.45 (33.10)	15 (57.50)	0 (23.21)	16.67 (62.50)

Note: Individual-level mean or median values were calculated first and then summarized into group-level mean or median values. * 95.6% and 96.0% of participant-reported activities were categorized into ACS activity domains for post-stroke and healthy groups, respectively; 4.4% and 4.0% activities were categorized as “other activities” for groups. Activities that did not match the ACS-based list were excluded.

**Table 3:** Objective measurements of affected arm use for self-reported activity participation categories and for upper limb functional capacities of affected arm of post-stroke group, as categorized using the Action Research Arm Test (ARAT).

ARAT	Activity Category (Activity Card Sort Version 3)				
Functional Capacity (Affected Arm)	Activities of Daily Living	Instrumental Activities	Leisure Activities	Social Activities	Fitness/Health Activities
Amount of arm use (vector magnitude)	Median (IQR)				
Low	0.00 (0.40)	0.00 (3.70)	0.00 (0.22)	2.27 (2.66)	0.55 (2.05)
Moderate	0.00 (1.79)	1.83 (18.19)	0.00 (0.37)	2.47 (2.98)	1.56 (22.75)
Good	0.00 (2.08)	9.67 (8.27)	0.00 (0.40)	6.65 (1.07)	**
Duration of arm use (minutes)	Mean ± SD				
Low	2.56 ± 2.15	2.94 ± 2.52	2.16 ± 1.22	3.61 ± 2.13	4.12 ± 1.74
Moderate	3.75 ± 2.66	3.95 ± 2.46	3.00 ± 1.49	4.56 ± 2.09	4.03 ± 2.06
Good	4.00 ± 0.00	4.71 ± 0.90	2.77 ± 0.58	6.06 ± 0.51	**
Perceived skill (z-score)	Median (IQR)				
Low	0.19 (0.66)	0.23 (1.72)	−0.45 (1.03)	−0.46 (0.47)	0.54 (1.44)
Moderate	−0.16 (1.00)	−0.20 (0.76)	−0.31 (0.60)	0.07 (0.60)	0.28 (0.57)
Good	−4.70 (0.00)	−0.32 (0.39)	0.14 (0.07)	1.54 (1.47)	**
Perceived challenge (z-score)					
Low	−0.46 (0.40)	0.36 (0.70)	−0.51 (1.03)	0.09 (0.85)	0.92 (0.59)
Moderate	0.39 (1.42)	0.56 (0.66)	−0.69 (0.58)	−0.49 (0.59)	0.87 (0.88)
Good	−4.00 (0.00)	−0.10 (0.35)	−0.10 (0.35)	1.07 (0.82)	**

Note: No valid data samples found for fitness/health activities for good capacity group. ** No data available.

**Table 4:** Effect of motor capability and psychosocial factors on affected arm use in everyday activity engagement.

		Model 1	Model 2	Model 3
Predictors		Coefficient (95% CI)		
Motor capability	ARAT	0.026 (0.002–0.050) *	0.2 (−0.006–0.045)	0.018 (−0.011–0.046) ^†^
Activity	ESM: ACS: leisure		−1.525 (−2.974–−0.076) *	
Social context	ESM: alone		−0.263 (−1.257–0.283)	−0.378 (−1.250–0.495)
Environment	ESM: at home		−0.689 (−1.010–0.484) *	−1.075 (−1.929–−0.220) *
Self-efficacy	ESM: self-efficacy		0.025 (−0.484–0.535)	
Perceived challenge	ESM: challenge		−0.24 (−0.409–0.361)	
Constant		2.798 (2.023–3.573) **	4.5 (2.975–6.026) **	3.795 (2.7709–4.880) **
Pseudo R^2^		0.052	0.254	0.311

Model 1 = motor capacity; Model 2 = Model 1 + activity context + psychosocial factors; Model 3 = simplified model with motor capacity and context factors. * *p* < 0.05, ** *p* < 0.001, ^†^ ARAT × ESM: at home 2-way interaction *p* < 0.005.

## Data Availability

The data are not publicly available due to planned further analyses.

## Supplementary Materials

The following supporting information can be downloaded at: https://www.mdpi.com/article/10.3390/s25237330/s1. Table S1: Participant-reported top six activities across groups and group-level perceived performances for post-stroke and healthy groups. Figure S1: Stroke-affected arm use duration by activity domain (left) and activity context (right). Table S2: Participant-level frequency of engagement in the 50 most common activities reported by healthy participants (*n* = 403 samples). Table S3: Participant-level frequency of engagement in the 50 most common activities reported by the post-stroke group. (*n* = 443 samples). Table S4: List of additional activities included in the ACS version 3-based list developed for this study.

## Abbreviations

The following abbreviations are used in this manuscript:

ACS	Activity Card Sort
ADL	Activities of Daily Living
ARAT	Action Research Arm Test
CI	Confidence Interval
ESM	Experience Sampling Method
IADL	Instrumental Activities of Daily Living
NIHSS	National Institutes of Health Stroke Scale
UL	Upper Limb
VM	Vector Magnitude
