# Supplementary material for: Combined Individual Experience and Accelerometry Measurement of Upper Limb Use in Daily Activities in Real Time After Stroke
Source: Sensors (Basel). 2025 Dec 2;25(23):7330. doi: 10.3390/s25237330 (PMC12694002; doi:10.3390/s25237330)
Supplement: Supplementary file 1 [file sensors-25-07330-s001.zip › sensors-3954794-supplementary.pdf]

Supplementary Materials:

Table S1. Participant-reported top 6 activities across groups and group-level perceived performances for post-stroke and healthy groups.

Activity (ACS)	*n* (%)	Skill	Challenge	Flow *n* (%)	Activity and motivation	Self-efficacy	Duration of affected arm use (minutes)	Amount of affected arm use (VM)
		Perceived z-score Median (IQR)					Mean ± SD	Median (IQR)
**Eating**								
Post-stroke	24 (5.4%)	-0.45 (1.36)	-0.70 (0.68)	5 (20.8%)	0.04 (0.43)	0.08 (0.81)	3.58 ± 2.21	0.00 (3.44)
Healthy	18 (4.5%)	0.35 (1.54)	-0.62 (0.54)	2 (11.1%)	-0.12 (0.43)	0.10 (0.36)	5.87 ± 1.84	1.70 (29.29)
**Work**								
Post-stroke	24 (5.4%)	0.23 (0.88)	0.72 (1.02)	8 (33.3%)	0.33 (0.19)	-0.04 (0.56)	3.97 ± 2.77	1.62 (16.71)
Healthy	70 (17.4%)	0.62 (0.64)	0.76 (0.78)	22 (31.4%)	0.19 (0.28)	0.18 (0.45)	4.64 ± 1.94	1.50 (9.05)
**Cooking**								
Post-stroke	22 (5.0%)	-0.00 (1.23)	0.51 (1.16)	8 (36.3%)	0.20 (0.30)	0.40 (0.76)	5.38 ± 3.03	11.98 (22.29)
Healthy	27 (6.7%)	0.41 (1.17)	-0.32 (1.12)	5 (18.5%)	0.10 (0.51)	0.42 (0.64)	5.26 ± 2.62	14.08 (30.90)
**Rest and relaxation**								
Post-stroke	127 (28.7%)	-0.46 (1.36)	-0.75 (0.87)	17 (13.4%)	-0.17 (0.35)	0.05 (0.31)	2.28 ± 1.48	0.00 (0.00)
Healthy	67 (16.6%)	-0.39 (1.03)	-0.35 (1.04)	2 (3.0%)	-0.25 (0.34)	-0.03 (0.54)	3.62 ± 1.67	0.00 (2.97)
**Watch TV**								
Post-stroke	22 (5.0%)	-0.16 (0.92)	-0.75 (1.14)	9 (40.9%)	-0.18 (0.94)	-0.12 (0.44)	1.38 ± 1.04	0.00 (0.00)
Healthy	16 (4.0%)	-0.37 (0.81)	-0.86 (1.40)	2 (3.0%)	-0.46 (0.71)	-0.30 (0.68)	4.24 ± 2.03	1.20 (9.53)
**Walking**								
Post-stroke	21 (4.7%)	-0.01 (0.99)	0.76 (0.64)	8 (38.0%)	0.16 (0.38)	0.59 (0.57)	5.01 ± 2.17	1.25 (25.56)
Healthy	14 (3.5%)	-0.49 (1.24)	-0.50 (1.12)	4 (28.6%)	-0.05 (0.63)	0.22 (0.80)	6.97 ± 2.80	49.87 (74.45)

Note: VM – vector magnitude

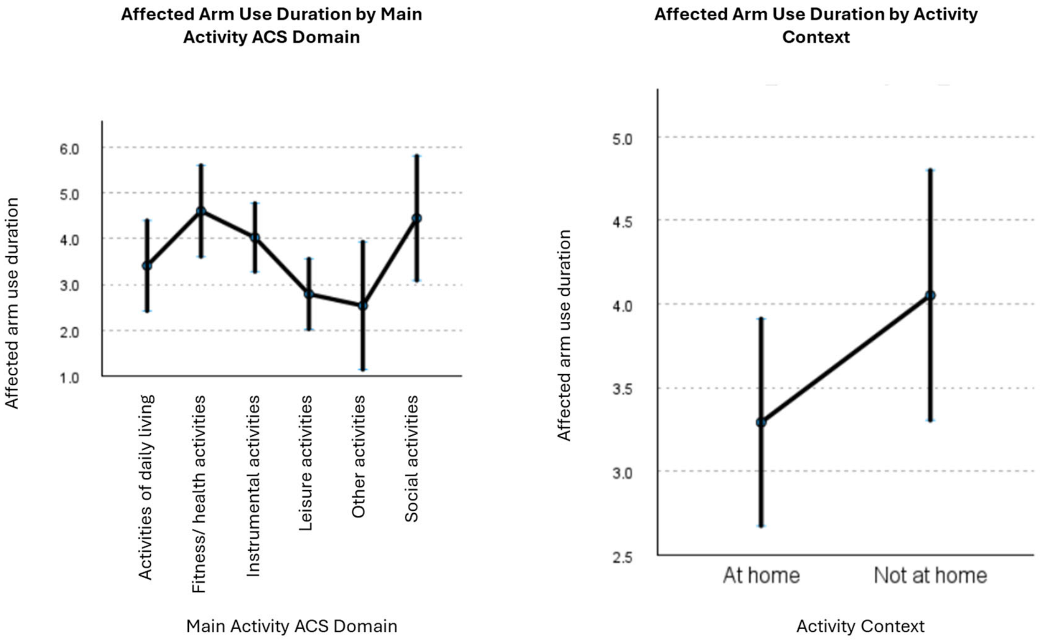

Figure S1. Stroke affected arm use duration by activity domain (left) and activity context (right). Values are estimated marginal means with a generalized linear mixed model for the top significant fixed effects of ACS activity domain and activity context (i.e., Model 2 of Table 4)

Table S2. Participant-level frequency of engagement in the 50 most common activities reported by healthy participants (*n*=403 samples).

Activity	Total samples (*n*)	Frequency Mean (SD)
Work	88	26.71 (17.21)
Rest and relaxation	111	25.87 (19.94)
Cooking	42	11.35 (7.46)
Email/social media	2	11.11
Eating	20	10.85 (6.45)
Reading magazines/books	29	9.09 (5.93)
Driving	14	8.07 (7.27)
Childcare	4	8.01 (0.45)
Other activity	21	7.79 (4.57)
Talking on the telephone	17	7.78 (3.47)
Watch TV	19	7.72 (6.66)
Light cleaning	14	7.69 (5.25)
Running	1	7.69
Shopping for groceries	19	7.55 (3.53)
Talking with family and neighbours	10	7.54 (4.18)
Gardening	3	7.42 (0.39)
Playing musical instruments	2	7.41
Horseback riding	1	6.67
Doing laundry	9	6.63 (2.73)
Walking	18	6.44 (3.48)
Reading religious material	2	5.95 (1.68)
Dressing	5	5.85 (2.87)
Spectator sports	3	5.84 (1.84)
Bathing	7	5.82 (1.82)
Taking care of plants	4	5.81 (3.63)
Pet care	8	5.76 (2.88)
Doing dishes	10	5.74 (1.85)
Yard maintenance	4	5.74 (3.28)

Reading newspaper	4	5.61 (2.06)
Taking transportation	4	5.56 (1.87)
Having a cuppa	1	5.56
Entertaining at home	1	5.56
Shopping for clothes/tools	1	5.56
Being with friends	5	5.52 (1.92)
Household projects	3	5.49 (2.33)
Tennis/golf/ other	4	5.19 (2.18)
Car maintenance	2	5.12 (1.09)
Swimming	6	5.05 (2.77)
Listening to radio	5	5.00 (2.03)
Yoga/pilates/Tai Chi	1	5.00
Go to movies	1	5.00
Heavy cleaning	1	4.76
Video games	1	4.76
Reading religious material	1	4.76
Go to park	2	4.73 (1.63)
Photography	2	4.73 (1.63)
Visiting family	3	4.65 (0.86)
Exercise	4	4.59 (0.72)
Text messaging	1	4.55

Note: Activities ranked from most common to least common. Participant-level frequency reported as mean and standard deviation. Activities based on Activity Card Sort version 3 [42]. SD – standard deviation.

Table S3. Participant-level frequency of engagement in the 50 most common activities reported by the post-stroke group. (*n*=443 samples).

Activity	Total samples (*n*)	Frequency Mean (SD)
Rest and relaxation	145	30.60 (19.88)
Pet care	2	21.67 (16.50)
Exercise	4	17.80 (13.39)
Work	25	17.03 (7.10)
Doing dishes	4	15.19 (15.79)
Eating	25	12.85 (8.36)
Browsing internet/shopping	9	12.49 (11.11)
Walking	22	11.96 (7.63)
Cooking	26	11.50 (8.55)
Bicycling	1	11.11
Mending/sewing clothes	1	11.11
S/Personal Advancement	2	11.11
Watch TV	27	11.06 (8.45)
Heavy cleaning	2	10.61 (8.57)
Shopping for groceries	6	10.56 (5.74)
Doing laundry	7	10.39 (7.37)
Therapy/exercises	25	10.25 (9.29)
Reading newspaper	1	10.00
Weight training	1	10.00
Driving for pleasure	1	9.09
Reading magazines/books	16	9.07 (4.54)
Other activity	22	8.96 (5.68)
Toileting	3	8.85 (1.63)
Taking transportation	10	8.78 (6.85)
Dressing	8	8.59 (4.64)
Childcare	7	8.58 (5.20)
Getting gas	2	8.39 (0.99)
Bathing	8	8.35 (2.93)

Creative writing	1	8.33
Window shopping	1	8.33
Household projects	1	7.69
Car maintenance	1	7.69
Shopping for clothes/tools	3	7.67 (2.01)
Listen to music	3	7.67 (2.01)
Eating out	3	6.85 (4.45)
Light cleaning	3	6.84 (2.73)
Driving	8	6.74 (3.44)
Listening to radio	2	6.67 (2.36)
Being with friends	3	6.30 (3.21)
Taking care of plants	3	6.20 (1.85)
Having a cuppa	4	5.96 (2.07)
Go to doctor	4	5.87 (2.79)
Spectator sports	4	5.82 (2.46)
Visiting family	1	5.56
Talking on the telephone	3	5.50 (0.66)
Study for school	2	5.30 (1.35)
Talking with family and neighbours	5	5.09 (2.86)
Sending letters or cards	2	5.05 (1.70)
Board games	3	5.03 (1.20)

Note: Activities ranked from most common to least common. Participant-level frequency reported as mean and standard deviation. Activities based on Activity Card Sort version 3 [42].

Table S4. List of additional activities included in the ACS version 3-based list developed for this study.

Activity	ACS Domain
Rest and relaxation	Leisure activities
Talking on the telephone	Social activities
Talking with family and neighbours	Social activities
Having a cuppa	Leisure activities
Toileting	Activities of daily living
Dressing	Activities of daily living
Bathing	Activities of daily living
Eating	Activities of daily living
Therapy/exercises	Fitness/ health activities

Note: The original 100 activities of ACS version 3 are not shown in this table. The final ACS-based activity list is presented in the manuscript.
